# Impact of Nutritional Status on Survival and Development of Hodgkin’s Lymphoma: A Scoping Review

**DOI:** 10.3390/nu17233777

**Published:** 2025-12-02

**Authors:** Sabina Krupa-Nurcek, Dominika Wiśniewska, Michał Klimas, Martyna Winiarska, Dominik Jucha, Arkadiusz Jamro

**Affiliations:** 1 Department of Surgery, Faculty of Medicine, Collegium Medicum, University of Rzeszow, 35-959 Rzeszow, Poland; 2 Faculty of Medicine, Collegium Medicum, University of Rzeszow, 35-959 Rzeszow, Poland; dominika.wisniewska.001@gmail.com (D.W.); michal.klimas02@gmail.com (M.K.); martynawiniarska44@gmail.com (M.W.); dominik.jucha125@gmail.com (D.J.); arekjamro@gmail.com (A.J.)

**Keywords:** Hodgkin’s lymphoma, nutritional status, survival, development

## Abstract

**Background/Objectives**: Hodgkin’s lymphoma (HL) is a cancer of the lymphatic system, the etiology of which remains partially unexplained, and environmental factors, including nutritional factors, may play an important role in its development and clinical course. The aim of this review was to examine the available literature on the impact of nutrition on the development and mortality of Hodgkin lymphoma. **Methods**: We conducted a literature review using databases, including publications from the last 10 years on nutrition and HL. Eventually, 3 publications were included in the review. **Conclusions**: Available data suggest that a diet rich in vegetables, fruits, fiber, and omega-3 fatty acids may have a protective effect, reducing the risk of developing Hodgkin’s lymphoma and improving prognosis and survival through anti-inflammatory and immune-supporting effects. On the other hand, excessive consumption of saturated fats, simple sugars and processed meat products can promote cancer transformation and worsen the course of the disease. Despite the promising results, further, well-designed prospective and interventional studies are needed to unequivocally determine the role of nutrition in the etiopathogenesis and treatment of HL.

## 1. Introduction

Hodgkin’s lymphoma (Latin: *Hodgkini lymphoma*; *HL*), which is one of the most common cancers of the lymphatic system, poses a significant clinical and epidemiological challenge, especially in the context of young adults [1,2]. Despite significant progress in diagnosis and treatment, which has led to a high cure rate, there is still a group of patients characterized by an unfavorable course of the disease and increased mortality [3,4]. In recent years, more and more attention has been paid to non-cancerous factors that can affect the prognosis, including the nutritional status of cancer patients. Nutrition, understood as a state of metabolic balance and availability of energy substrates, plays a key role in the immune response, treatment tolerance and tissue regeneration. In the context of Hodgkin’s lymphoma, which is characterized by an intense inflammatory response and often long-term cytotoxic treatment, eating disorders may be of particularly important prognostic importance [3].

In the scientific literature, it is increasingly emphasized that malnutrition, sarcopenia and metabolic disorders are independent risk factors for worsening treatment outcomes in cancer, including lymphomas. In the case of Hodgkin’s lymphoma, despite its relatively good prognosis, a significant percentage of patients with features of malnutrition is observed at the time of diagnosis [3,4]. This can be due to both general symptoms of the disease (such as fever, night sweats, weight loss) and chronic activation of the immune system and increased catabolism. Moreover, systemic treatment—including chemotherapy, immunotherapy and radiotherapy—can further exacerbate nutritional deficits by inducing nausea, vomiting, mucositis, or taste and appetite disorders [5,6,7,8].

The relationship between nutritional status and prognosis in Hodgkin lymphoma is multidimensional. On one hand, malnutrition can lead to a weakened immune response, increased susceptibility to infections and reduced treatment tolerance, which translates into the need to reduce doses or delay therapy [4,5,6]. On the other hand, the presence of sarcopenia—defined as loss of muscle mass and function—is associated with poorer treatment outcomes, regardless of body mass index (BMI). Retrospective and prospective studies have shown that patients with Hodgkin lymphoma and concomitant malnutrition have significantly lower rates of overall survival and progression-free survival, as well as a higher risk of treatment complications [8,9].

In the context of the above observations, the assessment of nutritional status should be an integral part of the diagnosis and treatment planning of patients with Hodgkin lymphoma. Tools such as BMI, Patient-Generated Subjective Global Assessment (PG-SGA) scale, electrical bioimpedance (BIA) measurements or computed tomography to assess muscle mass in a cross-section (e.g., at the L3 level) make it possible to identify patients at risk of malnutrition and sarcopenia. Early nutritional intervention—including both dietary support and oral or enteral supplementation—can improve treatment tolerance, reduce the risk of complications, and potentially improve cancer outcomes [10,11,12].

### 1.1. Nutritional Status in the Course of Cancer

**Figure 1:**
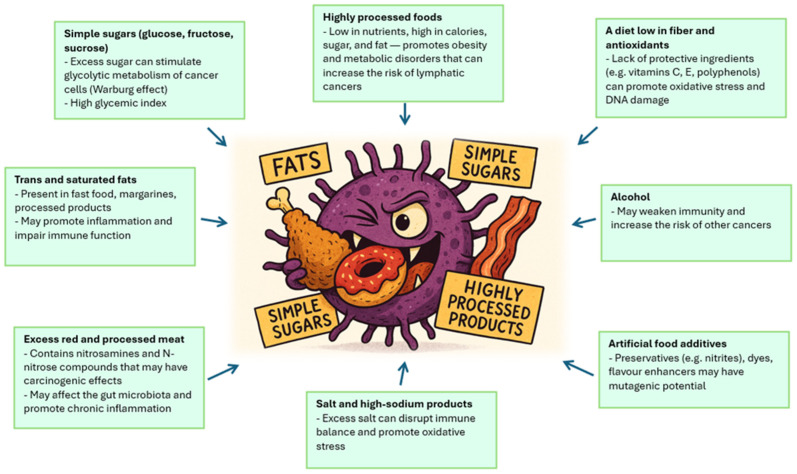
Food ingredients that influence the development of cancer. Source: Authors’ own work.

The patient’s nutritional status plays an important role in the course of cancer, including Hodgkin’s lymphoma, affecting both the development of the disease and the effectiveness of treatment and prognosis [13]. Hodgkin lymphoma, which is a tumor of the lymphatic system with a characteristic histopathological picture and immunophenotype, often occurs in young adults, and its clinical course can vary—from mild to aggressive. In this context, the patient’s nutritional status appears to be one of the key factors modifying the body’s response to the disease and treatment [14,15]. Malnutrition, understood as a deficiency in energy, protein and other nutrients, can occur as early as the diagnosis of Hodgkin’s lymphoma, especially in patients with general symptoms such as weight loss, fever or night sweats. These symptoms, which are part of the so-called B symptoms, are not only an indicator of disease activity, but can also lead to metabolic disorders and muscle catabolism. An overload of dietary sodium, phosphate, cholesterol, fat, and ultra-processed foods can play significant roles in dysregulating metabolism (affecting tissue growth and maintenance) and contributing to cancer risk and cachexia. As a consequence, nutritional status deteriorates, which may affect the functioning of the immune system, the ability to regenerate tissues and the tolerance of anticancer treatment [16]. Nutrition affects the development of Hodgkin lymphoma through several mechanisms. First, nutrient deficiency can weaken the immune response, which promotes disease progression and reduces the effectiveness of treatment. Second, malnutrition can lead to decreased muscle mass and sarcopenia, which are independent risk factors for a worse prognosis [17]. Sarcopenia, defined as loss of muscle mass and function, is associated with greater treatment toxicity, more frequent complications, and lower rates of overall survival and progression-free survival. In addition, treatment for Hodgkin lymphoma—including chemotherapy, immunotherapy and radiation therapy—may further exacerbate eating disorders [16,17,18]. Side effects of therapy, such as nausea, vomiting, mucositis, taste disorders or loss of appetite, lead to a decrease in food intake and a deterioration in the energy balance. As a result, there is a further deterioration in nutritional status, which may result in the need to modify treatment regimens, delays in therapy and an increased risk of infection and other complications. In light of the above observations, the assessment of nutritional status should be an integral part of the diagnosis and monitoring of patients with Hodgkin’s lymphoma [19,20]. Tools such as BMI, nutritional assessment scales (e.g., PG-SGA), bioimpedance measurements or analysis of muscle mass in computed tomography allow for the identification of patients at risk of malnutrition and sarcopenia. Early nutritional intervention, including dietary support, oral or enteral supplementation, can improve treatment tolerance, reduce the risk of complications, and potentially improve cancer outcomes [21]. Food ingredients that influence the development of cancer shows Figure 1.

### 1.2. Objectives and Rationale

The aim of this review was to examine the available literature on the impact of nutrition on the development and mortality of Hodgkin lymphoma. Of particular interest was information regarding nutrients that play a key role in the development of Hodgkin lymphoma and the impact of these nutritional factors on patient survival. The review question (RQ) for this review was: Does nutritional status influence the development of Hodgkin lymphoma and patient survival? The authors aimed to analyze nutritional factors that influence the development and mortality of Hodgkin lymphoma.

## 2. Materials and Methods

### 2.1. Study Design

The authors chose a scoping review because their goal was to examine concepts related to nutrition in Hodgkin lymphoma. Scoping reviews are rarely used in the literature, but they are a method that enhances the value of the review. There is little information in the literature on how to conduct a scoping review because there are few studies using this methodology [22]. This review was prepared according to the methods described in the Joanna Briggs Institute manual. This manual addresses scoping reviews and incorporates recommendations from the Preferred Reporting Items for Systematic Reviews and Meta-analysis for Scoping Reviews (PRISMA-ScR) guidelines [23,24].

### 2.2. Inclusion and Exclusion Criteria

The authors developed research questions that helped identify elements related to nutritional management in patients with Hodgkin lymphoma. Additionally, inclusion and exclusion criteria were established, as described below.

Inclusion criteria: articles from the last 10 years; all article types (original, observational and randomized studies, meta-analyses, systematic and narrative reviews); full-text articles; and articles in English.

Exclusion criteria: publications published before 2015; lack of access to the full text; and articles not in English.

#### 2.2.1. Population

The authors created a narrative review that included studies describing nutritional status in Hodgkin lymphoma and its impact on disease progression and mortality. In this review, nutritional status was defined as the quality or quantity of nutrients in food that indicates how well it may contribute to an individual’s health and well-being. Hodgkin lymphoma was defined as a cancer of the lymphatic system that develops from abnormally multiplying B lymphocytes. Its characteristic feature is the presence of giant cancerous Reed–Sternberg cells in the inflammatory infiltrate, which is also formed by other cells of the immune system, such as lymphocytes, monocytes and macrophages [1,2,3,17,18,19,20].

#### 2.2.2. Concept

The focus was on nutrition and its impact on the progression of Hodgkin lymphoma and patient mortality. The aim of this review was to examine the available literature on the impact of nutrition on the development and mortality of Hodgkin lymphoma.

#### 2.2.3. Context

The authors included studies that addressed nutrition in Hodgkin lymphoma.

#### 2.2.4. Types of Studies

The authors’ review included observational studies and one review with a methodology adapted to the project.

### 2.3. Search Strategy

Six authors searched available databases such as PubMed, Scopus, EBSCO, Web of Science, Google Scholar and Cochrane Library. Keywords used in the search: “Hodgkin lymphoma”, “Hodgkin lymphoma mortality”, “Hodgkin lymphoma nutrition”. Keyword combinations are introduced using the AND or OR operators. Initially, the articles were searched by analysing the abstracts of the publications. The authors have consulted several times on the publications that should be included. Each author independently analysed the inclusion criteria without prior consultation, and then all authors decided to make a final decision on the papers included in the review. The initial search ran from the beginning to 20 July 2025, and the final search was conducted on 20 September 2025.

### 2.4. Extraction of Data

For this review, the authors used a data extraction form based on the JBI Scope Review Guidelines [23]. This form included information from the included studies [24]. The authors analyzed the data from the studies. A Population–Concept–Context (PCC) model was used to identify relevant studies. The authors strictly followed the guidelines and the information regarding inclusion and exclusion criteria.

### 2.5. Critical Appraisal Process

A scoping review may include a review of current evidence without including a methodological assessment of the included studies [23].

### 2.6. Process for Including Publications to the Review

**Figure 2:**
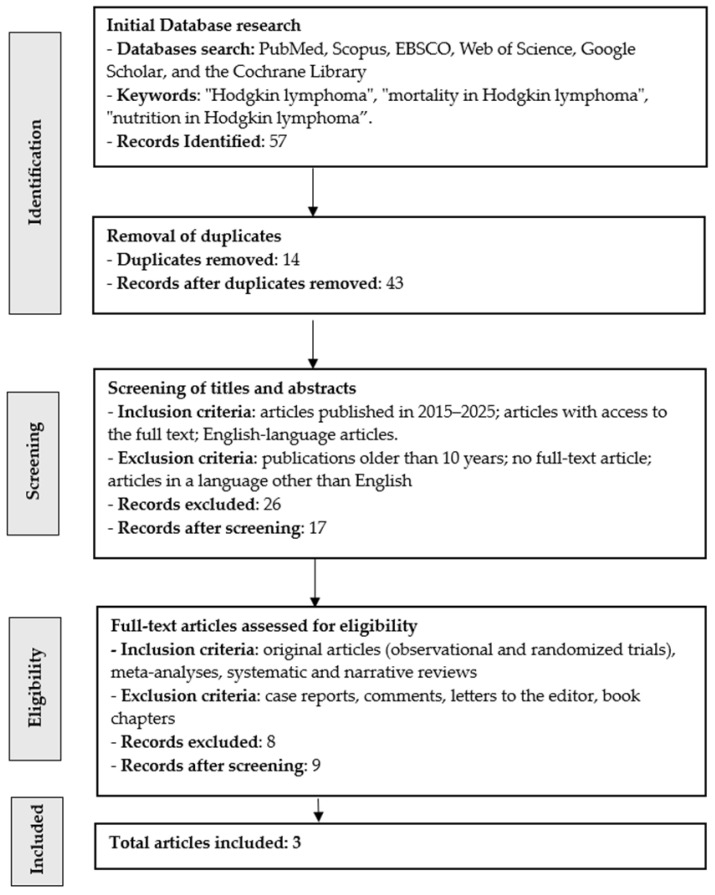
Flow chart for searching publications in this narrative review.

Our scoping review initially identified 57 articles, three of which were ultimately included in the analysis of nutritional factors influencing progression and mortality in Hodgkin lymphoma (Figure 2). After removing duplicates (n = 14), 43 articles remained. After reviewing the articles according to the inclusion and exclusion criteria (n = 26), 17 articles remained. Eight publications lacked full text and were excluded, leaving nine articles. As a result, after meeting all requirements, three publications were included in the review. The research was conducted in Pakistan (n = 1), Turkey (n = 1), Italy (n = 1). The results are presented in Table 1.

## 3. Nutritional Factors Influencing the Development and Mortality of Hodgkin Lymphoma

### 3.1. Excessive Consumption of Saturated Fats and Processed Meat Products

Among the nutritional components, excessive consumption of saturated fats and processed meat products is of particular importance, which can modulate inflammatory, immunological and metabolic processes that promote carcinogenesis [28]. Saturated fats, present mainly in animal products (e.g., red meat, butter, lard), exhibit pro-inflammatory potential by inducing the expression of cytokines such as interleukin-6 (IL-6) and tumor necrosis factor alpha (TNF-α). Chronic inflammation, which is a consequence of excessive consumption of these lipids, can lead to disorders in the functioning of the immune system, including deregulation of B and T lymphocytes, which play a key role in the pathogenesis of HL. In addition, saturated fats can affect the tumor microenvironment, promoting the proliferation of Reed–Sternberg cells—characteristic of Hodgkin’s lymphoma [29]. Processed meat products, such as cold cuts, sausages, bacon or canned meat, contain not only high amounts of saturated fats, but also chemical additives (e.g., nitrites, nitrates, preservatives), which under physiological conditions can be transformed into N-nitrose compounds with mutagenic activity. These compounds can damage the DNA of cells of the lymphatic system, initiating cancer transformation processes [28,29,30]. Furthermore, a diet rich in processed meat is associated with a lower intake of fiber, antioxidants, and protective nutrients, which further exacerbates the risk of developing HL [31]. Epidemiological studies indicate a correlation between high intake of saturated fat and processed meats and an increased risk of non-Hodgkin lymphoma and HL, especially in Western populations, where the “western diet” diet dominates over nutrition models based on vegetables, fruits, and whole grains [32]. It is worth noting that the impact of diet on HL risk may be particularly important during adolescence and early adulthood, when the immune system undergoes intense developmental changes. Excessive consumption of saturated fats and processed meat products may be a significant risk factor for the development of Hodgkin’s lymphoma through the induction of chronic inflammation, immune disorders and exposure to mutagenic compounds. In the context of HL prevention, it seems reasonable to promote a diet based on plant products, unsaturated fats and limiting the consumption of processed meat and animal fats [33,34].

High dietary phosphate intake causes the growth of lung and skin tumors in experimental animal models. Additional studies show that excessive phosphate loading induces pro-growth cell signaling, stimulates neovascularization, and is associated with chromosomal instability and metastasis. Studies have also shown that phosphate is a mitogenic factor that influences various tumor cell growth patterns, including hyperplasia in Hodgkin’s lymphoma [35].

### 3.2. High Glycemic Index and Excess of Simple Sugars

A high glycemic index (GI) and an excess of simple sugars in the daily diet may play an important role in modulating metabolic and immune processes that promote carcinogenesis. The glycemic index is a measure of the rate at which blood glucose levels rise after consuming a given carbohydrate-containing product. Products with a high GI (above 70) cause rapid spikes in glycaemia, which leads to intense insulin secretion and can promote the development of insulin resistance. Simple sugars, such as glucose, fructose or sucrose, present in sweets, carbonated drinks, highly processed products, are particularly disadvantageous due to their rapid absorption and strong effect on carbohydrate metabolism [36].

In the context of oncogenesis, excess simple sugars and high GI can affect the activation of metabolic pathways associated with cell proliferation, such as the insulin pathway and insulin-like growth factor IGF-1. IGF-1 is a known promoter of the growth of cancer cells, including lymphocytes, which provide the starting point for tumor transformation in HL [37]. In addition, chronic hyperglycemia can lead to oxidative stress, DNA damage and disruption of immune system cells. A high intake of simple sugars is also associated with an increase in chronic inflammation. Glucose and fructose can induce the expression of pro-inflammatory cytokines such as interleukin-6 (IL-6) and tumor necrosis factor alpha (TNF-α), which play a role in the tumor microenvironment. Chronic inflammation promotes deregulation of the immune response, which can lead to uncontrolled proliferation of lymphoid cells and their tumor transformation [38]. There is also data suggesting an association between a high-GI diet and an increased risk of hematologic cancers, including non-Hodgkin lymphomas. Although direct research on HL is limited, biological mechanisms point to the possibility of similar relationships. In particular, overweight and obese people who frequently consume foods with a high GI show an increased risk of developing lymphatic cancers [39]. Adipose tissue acts as an endocrine organ, secreting adipokines and pro-inflammatory cytokines, which can modulate the immune environment and promote carcinogenesis. It is also worth noting that a diet rich in simple sugars is often associated with a deficiency in nutrients with protective effects, such as fiber, antioxidant vitamins (C, E), polyphenols or minerals (magnesium, zinc). Deficiency in these substances can weaken DNA repair mechanisms, cellular immunity and the body’s ability to neutralize free radicals [40]. Excess simple sugars and a diet with a high glycemic index can be significant risk factors for the development of Hodgkin’s lymphoma by affecting glucose metabolism, insulin resistance, chronic inflammation and deregulation of the immune system. In the context of HL prevention, it is reasonable to promote a low-GI diet rich in whole grains, vegetables, fruits, and sources of fiber and antioxidants. This approach can not only reduce the risk of hematological cancers, but also support overall immunity and metabolic health [41,42].

### 3.3. Overweight and Obesity

Obesity, defined as a body mass index (BMI) ≥ 30 kg/m^2^, is a chronic, low-grade inflammatory condition that affects the functioning of the immune system, metabolism and the cellular microenvironment. Adipose tissue, especially visceral tissue, is not only a passive energy store, but an active endocrine organ, producing a number of bioactive substances—adipokines (e.g., leptin, adiponectin) and pro-inflammatory cytokines (e.g., TNF-α, IL-6, IL-1β) [43,44]. These mediators can influence the proliferation, survival, and transformation of lymphoid cells, which represents a potential pathogenetic mechanism in the development of HL. Leptin, the concentration of which increases in proportion to the amount of body fat, has pro-inflammatory and immunomodulatory effects. It can promote the proliferation of T and B lymphocytes and affect the expression of transcription factors associated with carcinogenesis such as NF-κB. On the other hand, the reduced levels of adiponectin—an anti-inflammatory adipokine—observed in obese people can lead to immune imbalances and promote the development of cancer [44]. Experimental studies have shown that altered concentrations of these adipokines can affect the tumor microenvironment, supporting angiogenesis, resistance to apoptosis, and tumor cell migration. Additionally, obesity is associated with insulin resistance and hyperinsulinemia, which can activate the insulin-like growth factor IGF-1 pathway. IGF-1 is a potent promoter of cell proliferation and an inhibitor of apoptosis, making it a potential mediator of cancer development, including lymphomas. High concentrations of IGF-1 can stimulate the growth of Reed–Sternberg cells—characteristic of HL—and support their survival in an unfavorable immune environment [45]. Epidemiological cohort and population-based studies provide evidence of an association between overweight and obesity and an increased risk of HL, particularly among young adults. Analyses of data from large databases such as the European Prospective Investigation into Cancer and Nutrition (EPIC) suggest that a higher BMI at an early age may correlate with a higher risk of developing HL later in life [43]. Importantly, this risk appears to be higher in men than in women, which may be due to hormonal and metabolic differences. It is also important that obesity affects the intestinal microbiota, whose disorders can modulate the immune response and promote chronic inflammation. Intestinal dysbiosis, observed in overweight individuals, can lead to increased intestinal barrier permeability and endotoxin translocation, which further intensifies the inflammatory response and may affect the risk of tumor transformation within the lymphatic system [46]. It is also worth noting that overweight and obesity often coexist with other adverse lifestyle factors, such as a high-calorie diet, low in fiber and antioxidants, low physical activity, and metabolic disorders (e.g., metabolic syndrome, type 2 diabetes) [47]. These factors may act synergistically, increasing the risk of developing HL by acting in multiple directions on the immune system, metabolism, and cellular environment. Overweight and obesity are important, modifiable risk factors for the development of Hodgkin lymphoma [48]. Their influence is realized through inflammatory, hormonal, metabolic and immune mechanisms that promote the transformation of cancer lymphoid cells. In the context of the prevention of HL and other hematological cancers, it is crucial to promote a healthy lifestyle, including a balanced diet, regular physical activity and maintaining a healthy body weight from an early age [49,50].

## 4. Effect of Nutritional Status on Mortality in Hodgkin Lymphoma

The nutritional status includes not only body weight, but also body composition (fat and muscle tissue ratios), the level of protein, vitamins, microelements and overall metabolic efficiency. Both malnutrition and obesity can negatively affect the course of HL [51]. Malnutrition, which often occurs in patients with advanced disease, is associated with weakened immunity, reduced tolerance to chemotherapy, a higher risk of infection, and impaired tissue regeneration. Obesity, on the other hand, although it may seemingly mask deficiencies, promotes chronic inflammation, insulin resistance and immune disorders, which can affect the effectiveness of cancer treatment [27]. Clinical trials have shown that patients with HL who have sarcopenia (loss of muscle mass) or protein–energy malnutrition have significantly worse treatment outcomes and a higher risk of death. Sarcopenia can lead to a decrease in metabolic reserve, making it difficult to tolerate intensive chemotherapy regimens such as ABVD (doxorubicin, bleomycin, vinblastine, dacarbazine). In addition, protein deficiency affects the synthesis of immunoglobulins, enzymes and cytokines, which weakens the immune response and increases susceptibility to opportunistic infections [52]. On the other hand, obesity can affect the pharmacokinetics of cytotoxic drugs, leading to their uneven distribution in the body. Adipose tissue can accumulate certain drugs, reducing their availability in target tissues. Additionally, obesity is associated with a higher risk of cardiovascular, thrombotic and metabolic complications, which can worsen the prognosis in HL. Retrospective studies have shown that patients with a BMI > 30 kg/m^2^ have a higher risk of death regardless of the stage of the disease, which may be due to the interaction between obesity and the tumor microenvironment [52,53]. It is also worth emphasizing the role of micronutrients and vitamins in the course of HL. Deficiencies in vitamin D, zinc, selenium or B vitamins can affect the functioning of the immune system, DNA repair processes and oxidative stress. Patients with HL are often deficient in these components, which can worsen the response to treatment and increase the risk of disease recurrence. However, supplementation should be individually adjusted, as an excess of certain substances (e.g., antioxidants) may interfere with the mechanism of action of chemotherapy. Nutritional assessment should be an integral part of the diagnosis and monitoring of patients with HL [54]. Tools such as body mass index (BMI), muscle mass measurement (e.g., by computed tomography), malnutrition rating scales (e.g., PG-SGA) and biochemical tests (albumin, prealbumin, CRP) allow for early detection of nutritional risks. Dietary interventions, nutritional support (e.g., oral, enteral, or parenteral nutrition), and metabolic rehabilitation can improve treatment tolerance, reduce the risk of complications, and improve survival [55,56]. The nutritional status of a patient with Hodgkin’s lymphoma has a significant impact on mortality and the course of the disease. Both malnutrition and obesity can worsen the prognosis by affecting immunity, metabolism and treatment tolerance. Incorporating nutritional assessment into cancer care standards and implementing appropriate interventions can be an important part of improving treatment outcomes and quality of life for patients with HL [57,58].

## 5. Protective Factors That Reduce the Risk of Development and Early Mortality in Hodgkin Lymphoma

### 5.1. A Diet Rich in Vegetables and Fruits

Vegetables and fruits are a source of numerous bioactive compounds, such as vitamins (C, E, K, FOLATE), minerals (potassium, magnesium, zinc), dietary fiber and polyphenols, carotenoids and flavonoids. These substances exhibit strong antioxidant properties, neutralizing free radicals formed as a result of oxidative stress, which accompanies both the process of cancer and cytotoxic treatment. Reduction in oxidative stress can protect healthy cells from damage, support DNA repair mechanisms, and reduce the risk of mutations that promote HL progression [59]. A diet rich in vegetables and fruits also affects the regulation of the immune response. Plant compounds such as sulforaphane (broccoli), quercetin (onions, apples), lycopene (tomatoes) or anthocyanins (berries) modulate the activity of T and B lymphocytes, support the function of natural killer (NK) cells and affect the expression of pro- and anti-inflammatory cytokines [60]. In the context of HL, where the tumor microenvironment is highly inflammatory, an immunomodulatory diet may limit the development of Reed–Sternberg cells and improve disease control. Another important aspect is the effect of a plant-based diet on the gut microbiota. Vegetables and fruits provide dietary fiber, which is a substrate for bacteria fermenting in the large intestine. Their metabolic products, such as short-chain fatty acids (SCFAs), exhibit anti-inflammatory effects, support the integrity of the intestinal barrier, and affect the immune system [61]. Microbiota disorders (dysbiosis), often seen in cancer patients, can increase the risk of infection, worsen treatment tolerance and negatively affect the prognosis. A diet rich in vegetables and fruits can restore microbial balance and support immune homeostasis. In the context of mortality, a plant-based diet may reduce the risk of metabolic complications such as insulin resistance, hyperlipidemia or hypertension, which may worsen the course of HL and increase the risk of death not directly related to cancer [62]. In addition, patients who follow a diet rich in vegetables and fruits show better tolerance to chemotherapy, a lower risk of malnutrition and faster recovery after treatment, which translates into improved overall health and a reduced risk of mortality. Population and observational studies indicate that people who follow a plant-based diet have a lower risk of developing hematological cancers, including lymphomas [63]. Although direct data on HL are limited, biological mechanisms and clinical observations support the hypothesis that a diet rich in vegetables and fruits may have a beneficial effect on the course of the disease and prognosis [64,65]. In particular, it is recommended to consume at least 400–600 g of vegetables and fruits per day, taking into account color diversity and seasonality, which provides access to a wide range of bioactive compounds. A diet rich in vegetables and fruits may play a vital role in reducing the risk of disease progression and mortality in Hodgkin lymphoma [10]. Its anti-inflammatory, antioxidant, immunomodulatory and supporting the intestinal microbiota make it an important element supporting oncological treatment. Incorporating nutritional recommendations into comprehensive HL patient care can improve quality of life, therapy tolerance, and long-term prognosis [66].

### 5.2. Dietary Fiber Intake

Dietary fiber, mainly present in plant products such as vegetables, fruits, whole grains, legumes and nuts, exhibits a number of biological properties that can affect the course of HL. Its effects include regulating the intestinal microbiota, modulating the immune response, reducing inflammation and improving metabolic parameters. High fiber intake is associated with a beneficial effect on immune homeostasis, which is particularly important in the context of lymphoid cancers, where immune system deregulation plays a key role in pathogenesis [67]. One of the main mechanisms of action of fibre is its effect on the gut microbiota. The fermentation of fiber by gut bacteria leads to the production of short-chain fatty acids (SCFAs) such as butyrate, propionate, and acetate. SCFAs have anti-inflammatory effects, support the integrity of the gut barrier and modulate the function of immune cells, including regulatory T cells [68]. Microbiota disorders (dysbiosis), often seen in cancer patients, can increase the risk of infection, worsen treatment tolerance and negatively affect the prognosis. A diet rich in fiber can restore microbial balance and support the body’s defenses. Fiber also reduces chronic inflammation, which is a recognized risk factor for cancer progression [69]. By lowering the level of pro-inflammatory cytokines (e.g., IL-6, TNF-α) and reducing oxidative stress, fiber can limit the proliferation of cancer cells and support the mechanisms of apoptosis. In the context of HL, where the tumor microenvironment is highly inflammatory, nutritional interventions with anti-inflammatory effects may be of significant clinical importance. High fiber intake is also associated with improved metabolic parameters such as glycemia, insulin resistance, lipid profile, and body weight [70]. Patients with HL often experience treatment-related metabolic disorders (e.g., steroid therapy, chemotherapy) that can increase the risk of complications and mortality. Fiber, by regulating the absorption of glucose and lipids, can support metabolic control and reduce the risk of metabolic syndrome, which is a worsening factor in the prognosis of cancer. Epidemiological studies indicate that people who follow a diet rich in fiber have a lower risk of developing cancers of the digestive and hematological systems [71]. Although direct data on HL are limited, biological mechanisms and clinical observations support the hypothesis that fiber may benefit the course of the disease and reduce the risk of death. In particular, it is recommended to consume a fibre intake of at least 25–35 g per day, taking into account a variety of plant sources. It is also worth noting that a diet rich in fiber often coexists with a high intake of antioxidants, vitamins and minerals that support immunity and tissue regeneration [72]. This nutritional approach can improve treatment tolerance, reduce the risk of malnutrition and support cell repair processes, which translate into a better prognosis and lower mortality. High dietary fiber intake may play a vital role in reducing the risk of disease progression and mortality in Hodgkin lymphoma. Its anti-inflammatory, immunomodulatory, metabolic and intestinal microbiota effects make it an important element supporting oncological treatment. Incorporating dietary recommendations for fiber into comprehensive care for a patient with HL may improve quality of life, therapy tolerance, and long-term prognosis [73].

### 5.3. Omega-3 Fatty Acids

Omega-3 fatty acids, such as eicosapentaenoic acid (EPA) and docosahexaenoic acid (DHA), are mainly found in oily marine fish (salmon, mackerel, sardine) and in fish and algae oils. Their anti-inflammatory effect is realized by inhibiting the synthesis of pro-inflammatory eicosanoids (e.g., prostaglandins series 2, leukotrienes series 4) and promoting the production of soluble mediators with anti-inflammatory activity, such as resolvins and protectins [74]. In the context of HL, where the tumor microenvironment is highly inflammatory, the presence of omega-3 may limit the activity of cytokines such as IL-6, TNF-α or IL-1β, which promote the proliferation of Reed–Sternberg cells and the progression of the disease. Omega-3 acids also affect the functioning of the immune system. Studies have shown that EPA and DHA modulate the activity of regulatory T cells, NK cells, and macrophages, supporting the anticancer response [75]. Additionally, omega-3s can improve cell membrane integrity, which affects cell signaling and resistance to oxidative stress. In patients with HL whose immune system is weakened by disease and treatment, immune support through diet may be important to control the disease and reduce the risk of recurrence. An important aspect of the action of omega-3s is their effect on metabolism and treatment tolerance [76]. Cancer cachexia syndrome, characterized by loss of muscle mass, malnutrition and metabolic disorders, is often observed in oncological patients. Omega-3s can counteract cachexia by inhibiting muscle protein catabolism, improving appetite, and supporting protein synthesis. The European Society for Clinical Nutrition and Metabolism (ESPEN) recommends omega-3 supplementation in cancer patients, especially when at risk of malnutrition. Clinical studies indicate that omega-3 supplementation can improve the quality of life of patients with hematological cancers, reduce side effects of chemotherapy (e.g., neuropathies, mucosal inflammation), and support tissue regeneration [77,78]. Although direct data on HL are limited, biological mechanisms and observations from other types of lymphomas and gastrointestinal cancers suggest that omega-3s may have a beneficial effect on the course of the disease and reduce the risk of death [79]. It is also worth noting that a diet rich in omega-3 often coexists with a high intake of other ingredients with protective effects, such as vitamin D, antioxidants and fiber. Such a nutritional approach can support immune homeostasis, improve treatment tolerance, and reduce the risk of metabolic complications, which are a significant factor in mortality in HL [80]. Omega-3 fatty acids may play a vital role in reducing the risk of disease progression and mortality in Hodgkin lymphoma. Their anti-inflammatory, immunomodulatory, metabolic and regenerative effects make them an important element supporting oncological treatment [81]. Incorporating omega-3 into a patient’s diet with HL, both through food and supplementation, can improve prognosis, quality of life, and long-term survival [79,82]. Summary of key nutritional factors influencing progression and mortality in Hodgkin lymphoma shows Table 2.

## 6. Limitations and Future Research

Scientific studies on nutrition in Hodgkin’s lymphoma are limited by the small number of prospective analyses, the heterogeneity of the study population, and the difficulty in unambiguously determining the impact of diet on the course of the disease. They are often based on observational data that do not allow a cause-and-effect relationship to be established between nutrients and HL risk. In addition, the lack of standardization of methods for assessing the state of nutrition and food intake makes it difficult to compare results between studies. Many analyses do not take into account the impact of treatment, co-morbidities, or genetic factors that may modify the body’s response to nutritional interventions. Further clinical trials with control of confounding variables are necessary to accurately determine the role of diet in the etiology and prognosis of Hodgkin lymphoma. Other research studies on the effects of nutrition on mortality in Hodgkin lymphoma are limited by the small number of prospective analyses and the difficulty of controlling for confounding variables such as disease stage, type of treatment, and patient metabolic status. They are often based on observational or retrospective data, which do not allow for a clear cause-and-effect relationship between diet and survival. There is also a lack of standardized methods for assessing nutritional status and nutrient intake, making it difficult to compare results between studies. In addition, many analyses do not take into account the impact of latent malnutrition (e.g., sarcopenia) or the quality of diet in the context of the gut microbiota and immunity. Further, well-designed clinical trials are needed to take a comprehensive approach to nutrition as a prognostic factor in HL.

In order to reliably assess the impact of nutrition on the development of Hodgkin lymphoma, it is necessary to undertake multi-stage scientific studies of high methodological quality, taking into account the complexity of the etiology of this disease and the interactions between diet, immune system and tumor microenvironment. Prospective cohort studies with long-term follow-up of large populations are crucial, in which dietary patterns, intake of specific nutrients and their correlation with the risk of developing HL will be analyzed in detail. Randomized controlled trials (RCTs) are also necessary, in which dietary interventions are compared for effects on inflammatory, immune, and metabolic biomarkers in at-risk individuals or patients with pre-disease conditions. This should be complemented by translational studies, including the analysis of the influence of dietary components on gene expression, lymphocyte function and cytokine profile, as well as studies of the intestinal microbiota as a mediator of nutritional effects. It is also important to develop standardised tools for assessing diet quality and nutritional status that allow for cross-site comparability of outcomes and the integration of clinical, nutritional and molecular data within multicentre trials.

## 7. Conclusions

The review work indicates that nutrition plays a significant role in both the risk of development and the course and mortality of Hodgkin lymphoma. Excessive consumption of saturated fats, simple sugars and processed meat products can promote cancer transformation by inducing chronic inflammation, immune disorders and oxidative stress. On the other hand, a diet rich in vegetables, fruits, fiber and omega-3 fatty acids has anti-inflammatory and immunomodulatory effects, supporting the body’s defense mechanisms and improving treatment tolerance. The patient’s nutritional status, both before diagnosis and during therapy, affects the prognosis, and malnutrition or obesity may increase the risk of complications and death. Further clinical studies are needed to precisely determine the relationship between diet quality and treatment outcomes in HL.

Nutrition plays a significant role in the course of cancer, including Hodgkin’s lymphoma, influencing both disease progression, treatment efficacy, and patient mortality. The mechanisms linking nutritional factors to cancer are extremely complex and involve metabolic, immunological, and epigenetic interactions. Diet can modulate the risk of disease, the course of therapy, and prognosis, and its importance stems from its impact on the immune system, gut microbiota, cellular metabolism, and inflammatory processes. At the molecular level, dietary components influence the proliferation and apoptosis of lymphocytes, from which Hodgkin’s lymphoma originates. Excessive intake of saturated fats and simple sugars promotes chronic inflammation and insulin resistance, which can stimulate signaling pathways associated with cancer development. In turn, a diet rich in antioxidants, fiber, and omega-3 fatty acids supports DNA repair mechanisms, reduces oxidative stress, and modulates the immune response, potentially reducing the risk of disease progression. The gut microbiota, shaped by diet, influences immunomodulatory metabolites, which can either support or weaken the effectiveness of chemotherapy. Diet is particularly important during Hodgkin lymphoma treatment. Chemotherapy and radiotherapy lead to taste disturbances, loss of appetite, nausea, and diarrhea, which increase the risk of malnutrition. Malnutrition is a key factor in worsening prognosis and increasing mortality, as it weakens immunity, reduces treatment tolerance, and prolongs recovery time. A balanced diet providing adequate protein, energy, and micronutrients can improve treatment outcomes, reduce complications, and support bone marrow and immune system regeneration. After treatment, diet remains an important element in preventing relapses and improving quality of life. Eating habits influence the risk of secondary cancers and cardiovascular disease, which are common complications following Hodgkin lymphoma treatment. Proper nutrition supports metabolic balance, reduces chronic inflammation, and improves overall health, which translates into lower long-term mortality. Nutrition in Hodgkin lymphoma is not only a treatment-supporting element but also a factor modulating disease progression and prognosis. The complex mechanisms lie in the multi-level impact of diet on metabolism, immunity, microbiota, and inflammatory processes, making it an integral component of cancer therapy. A conscious approach to nutrition can reduce mortality, improve treatment efficacy, and increase the chances of lasting remission.

## Figures and Tables

**Table 1:** Characteristics and findings of studies included in this review.

Author, Year	Country	Participants	Findings
Ghafoor T. et al., 2019 [25]	Pakistan	Pediatric patients with Hodgkin lymphoma	✔ Early initiation of treatment improves prognosis ✔ In patients with a history of anti-tuberculosis treatment, early mortality was higher ✔ Nutritional status had an impact on the prognosis of patients with HL ✔ A delay in the start of treatment by more than one year was a significant adverse factor
Gürsoy V. et al., 2023 [26]	Turkey	Patients with Hodgkin lymphoma	✔ Nutritional assessment plays an important role in the response and survival of people with HL ✔ Malnutrition may reduce patients’ tolerance to chemotherapy and increase the risk of secondary infections ✔ Malnutrition is a potential independent prognostic factor for patients with HL
Mancuso S. et al., 2022 [27]	Italy	Patients with Hodgkin lymphoma	✔ The state of malnutrition works in synergy with the aggressiveness of the lymphoma in increasing the risk of treatment failure ✔ HL triggers a chain of pathological and inflammatory events that induce metabolic breakdown, causing malnutrition ✔ Malnutrition, sarcopenia, and weakness may be partly responsible for the biological characteristics of cancer and its development

**Table 2:** Summary of key nutritional factors influencing progression and mortality in Hodgkin lymphoma.

Factor	Key Findings	References
Excessive consumption of saturated fats and processed meat products	Excessive consumption of saturated fats and processed meat products may promote the development of Hodgkin’s lymphoma by inducing chronic inflammation, deregulating the immune system, and being exposed to mutagenic compounds present in canned meat. This type of diet can lead to disorders of the lymphoid microenvironment, increasing the risk of cancerous transformation of lymphatic cells.	[4,28,29,30,31,32,33,34,35]
High glycemic index and excess of simple sugars	A high glycemic index and excess simple sugars may promote the development of Hodgkin’s lymphoma by inducing insulin resistance, chronic inflammation, and activating the IGF-1 pathway, which stimulates the proliferation of cancer cells. In addition, a diet rich in refined sugars weakens immunity and promotes oxidative stress, which may increase the risk of lymphocyte transformation towards cancer.	[15,16,26,36,37,38,39,40,41,42]
Overweight and obesity	Overweight and obesity can increase the risk of developing Hodgkin’s lymphoma by inducing chronic inflammation, impaired immune function, and overproduction of pro-inflammatory cytokines by adipose tissue. Additionally, obesity promotes insulin resistance and deregulation of the IGF-1 pathway, which may promote lymphoid cell proliferation and tumor transformation.	[14,27,43,44,45,46,47,48,49,50]
A diet rich in vegetables and fruits	A diet rich in vegetables and fruits may reduce the risk of developing Hodgkin’s lymphoma thanks to the presence of antioxidants, fiber, and anti-inflammatory compounds that support immunity and protect cells from DNA damage. Regular consumption of these products promotes immune balance and reduces the influence of environmental factors that promote the transformation of cancer lymphocytes.	[10,26,27,59,60,61,62,63,64,65,66]
Dietary fiber intake	Dietary fiber intake may reduce the risk of developing Hodgkin’s lymphoma by supporting the gut microbiota, reducing chronic inflammation, and improving cellular immunity. In addition, fiber has a positive effect on metabolism and immune balance, limiting factors that promote the transformation of cancer lymphocytes.	[8,25,67,68,69,70,71,72,73]
Omega-3 fatty acids	Omega-3 fatty acids may reduce the risk of developing Hodgkin lymphoma through their anti-inflammatory, immunomodulatory and supportive effects on metabolic balance and the lymphoid microenvironment. Their presence in the diet promotes the reduction of cancer cell proliferation and improves immunity, which may protect against cancerous transformation of lymphocytes.	[74,75,76,77,78,79,80,81,82]

## Data Availability

Not applicable.

## Abbreviations

The following abbreviations are used in this manuscript:

HL	Hodgkin lymphoma
PG-SGA	Patient-Generated Subjective Global Assessment
BMI	Body Mass Index
BIA	Bioimpedance
RQ	Review Question
PRISMA-ScR	Preferred Reporting Items for Systematic Reviews and Meta-analysis for Scoping Reviews
IL-6	Interleukin-6
TNF	tumor necrosis factor
IG	Glycemic Index
IGF-1	Insulin-like growth factor-1
NF-κB	Nuclear factor kappa-light-chain-enhancer of activated B cells
EPIC	European Prospective Investigation into Cancer and Nutrition
ABVD	adriamycin, bleomynic, vinblastine, dacarbazine
CRP	C-reactive protein
NK-cells	natural-killer cells
SCFA	short-chain fatty acid
ESPEN	European Society for Clinical Nutrition and Metabolism
EPA	Eicosapentaenoic acid
DHA	Docosahexaenoic acid
RCT	randomized controlled trial
